# Graph-Enhanced Expectation Maximization for Emission Tomography

**DOI:** 10.3390/jimaging12010048

**Published:** 2026-01-20

**Authors:** Ryosuke Kasai, Hideki Otsuka

**Affiliations:** Institute of Biomedical Sciences, Tokushima University, 3-18-15 Kuramoto, Tokushima 770-8509, Japan; hideki.otsuka@tokushima-u.ac.jp

**Keywords:** image reconstruction, graph laplacian, maximum-likelihood expectation maximization, single-photon emission computed tomography

## Abstract

Emission tomography, including single-photon emission computed tomography (SPECT), requires image reconstruction from noisy and incomplete projection data. The maximum-likelihood expectation maximization (MLEM) algorithm is widely used due to its statistical foundation and non-negativity preservation, but it is highly sensitive to noise, particularly in low-count conditions. Although total variation (TV) regularization can reduce noise, it often oversmooths structural details and requires careful parameter tuning. We propose a Graph-Enhanced Expectation Maximization (GREM) algorithm that incorporates graph-based neighborhood information into an MLEM-type multiplicative reconstruction scheme. The method is motivated by a penalized formulation combining a Kullback–Leibler divergence term with a graph Laplacian regularization term, promoting local structural consistency while preserving edges. The resulting update retains the multiplicative structure of MLEM and preserves the non-negativity of the image estimates. Numerical experiments using synthetic phantoms under multiple noise levels, as well as clinical ^99m^Tc-GSA liver SPECT data, demonstrate that GREM consistently outperforms conventional MLEM and TV-regularized MLEM in terms of PSNR and MS-SSIM. These results indicate that GREM provides an effective and practical approach for edge-preserving noise suppression in emission tomography without relying on external training data.

## 1. Introduction

Image reconstruction in emission tomography, such as single-photon emission computed tomography (SPECT), aims at estimating an underlying radiotracer distribution from projection data that are inherently noisy and often incomplete [1,2,3,4]. This ill-posed nature necessitates reconstruction algorithms that are both numerically stable and physically meaningful, motivating extensive research on statistically grounded iterative methods [5].

The maximum-likelihood expectation maximization [6,7] (MLEM) algorithm has become a standard approach in emission tomography due to its sound Poisson noise modeling and its intrinsic preservation of non-negativity in image estimates. Despite these advantages, MLEM is well known to suffer from noise amplification as iterations proceed, especially in low-count regimes, which can severely degrade image quality in practical applications [8,9]. To alleviate this issue, various regularized EM-type methods have been proposed, including approaches based on total variation (TV) penalties. While TV regularization can effectively suppress noise and preserve edges, it often introduces excessive smoothing and requires careful parameter tuning, which may be nontrivial in clinical settings [10,11,12].

Recently, graph-based image processing techniques have attracted increasing attention in tomographic reconstruction [13]. By modeling pixel interactions through graph structures, these methods can incorporate spatial and intensity similarities beyond purely local neighborhoods, enabling edge-aware smoothing and improved robustness to noise. Graph-based regularization has been explored in various forms, including nonlocal filters [14], graph Laplacians, and graph-guided denoising applied either before or after reconstruction [15,16,17]. However, many existing approaches rely on additive gradient-based updates, alternating optimization schemes, or heuristic integration of graph operations, which may complicate the preservation of key properties inherent to EM-type algorithms, such as non-negativity of the iterates. Moreover, while general convergence results for EM and MM algorithms exist [18], they do not directly address the interplay between graph-structured penalties and multiplicative update schemes commonly used in emission tomography.

In this work, we propose a graph-enhanced expectation maximization algorithm, referred to as Graph-Enhanced EM (GREM), which integrates graph-based neighborhood information into an MLEM-type multiplicative iteration. The proposed method is motivated by a penalized objective function consisting of a Kullback–Leibler (KL) divergence [19] data-fidelity term and a quadratic graph Laplacian term constructed from local spatial proximity and intensity similarity. This formulation provides an intuitive criterion for encouraging consistency among strongly related neighboring pixels while allowing transitions across weakly connected regions. Importantly, the resulting update retains the multiplicative structure characteristic of MLEM, thereby preserving non-negativity of the image estimates under mild and standard assumptions.

From a theoretical standpoint, we establish a fundamental property of the proposed GREM iteration, namely the preservation of non-negativity of the image estimates throughout the reconstruction process. This property is essential for emission tomography, as reconstructed activity distributions must remain physically meaningful. Other desirable behaviors, such as improved noise robustness and edge preservation, are investigated through systematic numerical experiments rather than imposed as strict theoretical constraints.

The practical performance of the proposed method is evaluated using numerical phantom studies under varying noise levels, as well as clinical SPECT data acquired in a routine imaging setting. Quantitative metrics and visual assessments demonstrate that GREM consistently improves image quality compared with conventional MLEM and TV-regularized MLEM, especially in high-noise scenarios. These results indicate that incorporating graph-based neighborhood interactions into a multiplicative EM-type framework can offer a favorable balance between noise suppression and structural preservation without relying on external training data.

This study presents a graph-enhanced reconstruction framework that extends the practical capabilities of MLEM while maintaining its essential structural properties. The proposed approach provides a flexible and interpretable basis for further extensions, including adaptive graph construction and three-dimensional tomographic reconstruction.

## 2. Definitions and Notations

Tomographic image reconstruction fundamentally involves determining an unknown image $x\in R_{+}^{J}$ based on observed projection data $p\in R^{I}$, often modeled by the following linear relationship:

(1) p=Hx+σ,

where $H\in R^{I\times J}$ is the system matrix that describes the mapping from image space to projection space based on the imaging geometry, and $\sigma \in R^{I}$ denotes the measurement noise [20,21]. Here, R denotes the set of real numbers, and $R_{+}$ denotes the set of non-negative real numbers. We further denote by $R_{++}$ the set of strictly positive real numbers.

In tomographic reconstruction, the matrix *H* is determined by the physical setup of the imaging system, including the detector configuration and the spatial sampling of the image domain, thereby encoding the geometric relationship between the object being imaged and the acquired projections. In practice, the inverse problem described by Equation (1) is often ill-posed due to factors such as limited-angle data acquisition, photon attenuation, and Poisson noise in the measured counts. Small perturbations in *p* can lead to large deviations in the solution *x* when naively inverting *H*, highlighting the sensitivity of direct inversion to noise and incomplete data [22].

## 3. Maximum-Likelihood Expectation Maximization Algorithm

The MLEM algorithm is a widely used iterative method for tomographic image reconstruction, particularly in emission computed tomography. It is derived from the statistical modeling of photon detection as a Poisson process and can be interpreted as a minimization of the KL–divergence between the measured projection data and the data predicted from the current image estimate.

Let the measured projection data be denoted as $p=\{p_{1},p_{2},\ldots ,p_{I}\}$, and the image to be reconstructed as $x=\left\{x_{1},x_{2},\ldots ,x_{J}\right\}\in R_{+}^{J}$. The system matrix $H=\left\{h_{ij}\right\}\in R^{I\times J}$ models the imaging process, where $h_{ij}$ denotes the contribution of pixel *j* to detector bin *i*. The expected projection at bin *i* is given by ${\left(Hx\right)}_{i}=\sum_{j=1}^{J}h_{ij}x_{j}$.

The KL–divergence between the measured data *p* and the expected data Hx is defined as:

(2) $$\mathrm{KL}\left(p,Hx\right)=\sum_{i=1}^{I}p_{i}\log\frac{p_{i}}{{\left(Hx\right)}_{i}}-p_{i}+{\left(Hx\right)}_{i}.$$

Since $\sum_{i}p_{i}\log p_{i}$ does not depend on *x*, it can be omitted from the optimization, leading to the following equivalent objective function:

(3) $$\sum_{i=1}^{I}{\left(Hx\right)}_{i}-p_{i}\log{\left(Hx\right)}_{i}.$$

Taking the partial derivative with respect to $x_{j}$, we obtain

(4) $$\frac{\partial }{\partial x_{j}}\mathrm{KL}\left(p,Hx\right)=\sum_{i=1}^{I}h_{ij}\left(1-\frac{p_{i}}{\sum_{j^{\prime }=1}^{J}h_{ij^{\prime }}x_{j^{\prime }}}\right).$$

Setting this derivative to zero leads to the following condition:

(5) $$\sum_{i=1}^{I}h_{ij}\cdot \frac{p_{i}}{\sum_{j^{\prime }=1}^{J}h_{ij^{\prime }}x_{j^{\prime }}}=\sum_{i=1}^{I}h_{ij}.$$

This condition cannot be solved analytically in general. Instead, the MLEM algorithm satisfies this balance through an iterative multiplicative update rule:

$$z_{j}\left(k+1\right)=z_{j}\left(k\right)\left(f_{j}\left(z\left(k\right)\right)\right),\;z\left(0\right)=x^{0}\in R_{++}^{J},$$

where the iterative function $f_{j}\left(x\right)$ is defined as follows:

(6) $$f_{j}\left(x\right):=\frac{1}{\sum_{i=1}^{I}h_{ij}}\sum_{i=1}^{I}h_{ij}\frac{p_{i}}{\sum_{j^{\prime }=1}^{J}h_{ij^{\prime }}x_{j^{\prime }}}.$$

This update is repeated over iterations k=0,1,2,…,K−1, with an initial estimate $x^{0}\in R_{++}^{J}$. At each step, the image is updated based on the ratio of the measured projection data *p* to the estimated projection Hx, ensuring monotonic reduction of the KL–divergence and convergence to a stationary point under standard assumptions for the MLEM algorithm.

## 4. Proposed Algorithm

While the MLEM algorithm is widely used due to its statistical foundation and non-negativity preservation, it is highly sensitive to noise in the projection data, often leading to noise amplification as iterations proceed. In addition, standard MLEM does not explicitly exploit spatial or intensity correlations among neighboring pixels, which are important for preserving structural coherence in practical tomographic imaging.

To mitigate these limitations, we propose a graph-enhanced multiplicative extension of MLEM that incorporates spatial and intensity similarities through a graph-based coupling term. The proposed update retains the multiplicative structure of MLEM and therefore preserves non-negativity of the image estimates under standard assumptions. Moreover, the update reduces exactly to the conventional MLEM iteration when the graph regularization weight is set to zero.

The formulation is guided by a penalized likelihood criterion involving a graph Laplacian term, which serves as a principled design guideline rather than a strictly optimized global objective. This allows adaptive neighborhood interactions to be incorporated into an EM-type multiplicative iteration while maintaining the essential properties of MLEM.

### 4.1. Objective Function Formulation for GREM

We refer to this graph-enhanced multiplicative extension of MLEM as Graph-Enhanced EM (GREM). To motivate the incorporation of graph-based regularization, we consider the following penalized objective function:

(7) $$J\left(x\right):=\mathrm{KL}\left(p,Hx\right)+\frac{\gamma }{2}\;x^{⊤}Lx,$$

where the first term represents the Poisson data-fidelity measured by the KL–divergence, and the second term is a quadratic graph-based regularization.

The graph Laplacian L=D−W is constructed from an 8-connected neighborhood system over the image domain. The edge weight between pixels *s* and *t* is defined by

(8) $$W_{st}=\exp\;\left(-\frac{∥d_{s}-d_{t}{∥}_{2}^{2}}{2\epsilon_{d}}\right)\exp\;\left(-\frac{∥v_{s}-v_{t}{∥}_{2}^{2}}{2\epsilon_{r}}\right),$$

where $d_{s}$ and $d_{t}$ denote the spatial coordinates of pixels *s* and *t*, and $v_{s}$ and $v_{t}$ denote their corresponding pixel intensity values. The parameters $\epsilon_{d}$ and $\epsilon_{r}$ control the sensitivity to spatial proximity and intensity similarity, respectively.

The corresponding degree matrix is given by $D=\mathrm{diag}(d_{1},\ldots ,d_{J})$ with $d_{j}=\sum_{j^{\prime }}W_{jj^{\prime }}$, yielding the non-negative quadratic form

(9) $$x^{⊤}Lx=\frac{1}{2}\sum_{j,j^{\prime }}W_{jj^{\prime }}{\left(x_{j}-x_{j^{\prime }}\right)}^{2}\geq 0,$$

which promotes local consistency while allowing intensity transitions across weakly connected regions.

### 4.2. Multiplicative Update Rule

To obtain a practical reconstruction algorithm, we adopt an iterative multiplicative update that extends the standard MLEM correction by introducing a graph-based coupling term. The update is constructed so as to preserve the multiplicative EM-type structure of MLEM, which guarantees non-negativity of the image estimates, while incorporating additional neighborhood interactions to improve robustness against noise.

The data-fidelity term KL(p,Hx) yields the standard MLEM correction factor based on the ratio between the measured projection data and the current estimated projections. In addition, the graph term introduces Laplacian-type interactions among neighboring pixels through L=D−W, with the regularization parameter γ controlling the strength of the graph-based coupling. In the proposed method, the graph is constructed at each iteration based on the current image estimate.

Combining these two components results in the following multiplicative update:

$$z_{j}\left(k+1\right)=z_{j}\left(k\right)\;g_{j}\left(z\left(k\right)\right),\;z\left(0\right)=x^{0}\in R_{++}^{J},$$

where the update factor is defined by

(10) $$g_{j}\left(x\right):=\frac{\sum_{i=1}^{I}h_{ij}\frac{p_{i}}{{\left(Hx\right)}_{i}}\;+\;\gamma \sum_{j^{\prime }}W_{jj^{\prime }}\;x_{j^{\prime }}}{\sum_{i=1}^{I}h_{ij}\;+\;\gamma \;d_{j}\;x_{j}}.$$

The update rule is consistent with the penalized objective function (7) in the sense that it is obtained from a non-negative gradient decomposition, leading to a multiplicative EM-type iteration whose fixed points satisfy the associated stationarity conditions.

By construction, this multiplicative update preserves non-negativity of the iterates for any non-negative initialization. When γ=0, the update (10) reduces exactly to the conventional MLEM iteration. Compared with standard MLEM, the proposed update incorporates graph-based neighborhood information at each iteration, providing improved robustness to noise and enhanced preservation of structural features in the reconstructed image.

### 4.3. Non-Negativity Preservation

In this subsection, we establish a basic structural property of the proposed GREM iteration, namely the preservation of non-negativity of the image estimates. This property follows directly from the multiplicative form of the update and is essential in emission tomography, where the reconstructed activity distribution must remain physically meaningful.

**Proposition** **1.** *Assume that $h_{ij}\geq 0$ and $p_{i}\geq 0$ for all i,j, and that $c_{j}:=\sum_{i=1}^{I}h_{ij}>0$ for all j. Let γ≥0, $W_{jj^{\prime }}\geq 0$, and $d_{j}:=\sum_{j^{\prime }}W_{jj^{\prime }}$. Given an initial estimate $z\left(0\right)\in R_{+}^{J}$, the GREM iteration defined in Section 4.2 preserves non-negativity, i.e.,*

$$z\left(k\right)\in R_{+}^{J}\;\mathrm{for}\;\mathrm{all}\;k\geq 0.$$

**Proof.**  Assume $z\left(k\right)\in R_{+}^{J}$. Since the update is multiplicative and all terms involved in the update factor are non-negative, while the denominator is strictly positive due to $c_{j}>0$, it follows that $z_{j}\left(k+1\right)\geq 0$ for all *j*. The result follows by induction. □

From an asymptotic point of view, each GREM iteration has computational complexity O(nnz(H)+J), where nnz(H) denotes the number of nonzero elements in the system matrix *H*. Specifically, the forward and back projection operations scale as O(nnz(H)), while graph construction and Laplacian interactions scale as O(J) due to the fixed local neighborhood. As a concrete numerical example, for 128×128 images with 60×128 projection bins, we have J=16,384 and *I* = 7680, yielding $\mathrm{nnz}\left(H\right)\approx 9.8\times 10^{5}$. Under these conditions, the projection-related computations dominate the runtime, and the graph-related overhead remains moderate. Even with the increased execution time, the additional computational cost can be justified if it yields substantially improved reconstruction quality, as demonstrated by the enhanced noise suppression and edge preservation observed in the proposed method.

## 5. Experiments

To assess the performance of the proposed GREM algorithm, we conducted a series of experiments using simulated tomographic projection data under various noise conditions and phantom configurations. The performance was evaluated in comparison with two baseline methods under identical initialization and stopping criteria to ensure a fair comparison. The first is the standard MLEM algorithm, which reconstructs the image solely based on the likelihood model without incorporating any form of regularization. The second is a regularized variant of MLEM that includes a TV penalty term (referred to as MLEM-TV), which is widely used to preserve edge information while suppressing noise in the reconstructed image.

### 5.1. Experiments with Numerical Phantoms

To evaluate the reconstruction performance of the proposed method under controlled conditions, we conducted experiments using two well-known numerical phantoms: the Shepp–Logan phantom and the Digitized Hoffman phantom. The Shepp–Logan phantom is a standard benchmark for tomographic algorithms, characterized by smooth intensity variations and sharp edges, while the Hoffman 3D brain phantom (Acrobio Co., Ltd. Tokyo, Japan ) mimics realistic cerebral perfusion distributions with intricate anatomical features. These phantoms, each of size 128×128 pixels, serve as the ground truth images for quantitative comparison.

**Figure 1:**
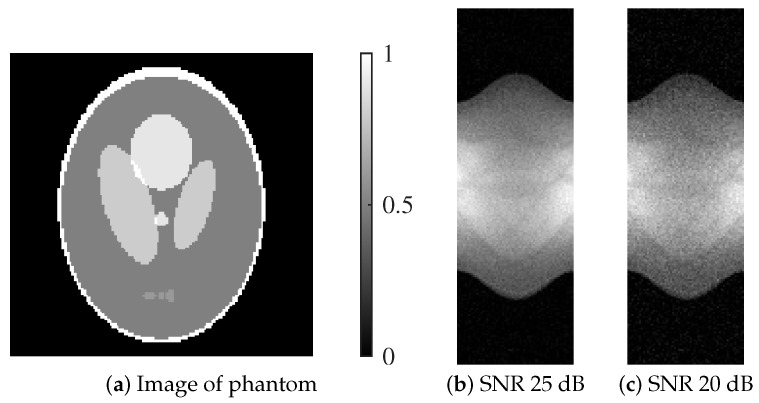
Shepp–Logan phantom utilized in the numerical experiments and their respective sinograms: (**a**) Image of phantom, (**b**) SNR 25 dB, (**c**) SNR 20 dB.

Figure 1 presents the Shepp–Logan phantom and its corresponding projection data under two different noise levels.

The projection data *p* were generated using a parallel-beam acquisition geometry with 60 projection angles uniformly distributed over 180 degrees. The system matrix *H* was generated accordingly. Poisson noise σ was added to simulate projection data at signal-to-noise ratios (SNRs) of 25 dB and 20 dB, representing moderate and high noise conditions.

**Figure 2:**
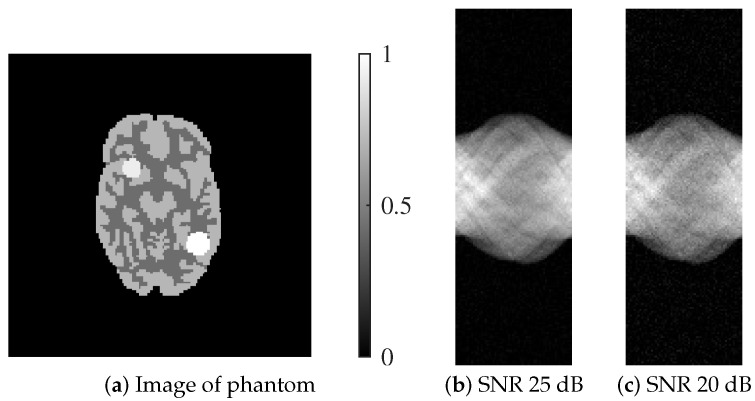
Digitized Hoffman phantom utilized in the numerical experiments and their respective sinograms: (**a**) Image of phantom, (**b**) SNR 25 dB, (**c**) SNR 20 dB.

Similarly, Figure 2 displays the digitized Hoffman phantom alongside its simulated projection data at the same two noise levels. The phantom was created by extracting a representative slice from the 3D Hoffman brain phantom, where brain parenchyma was modeled with signal intensities of 0.3 and 0.5. In addition, simulated radiotracer uptake was introduced by placing two regions with higher intensities: 0.65 in the upper right and 0.7 in the lower left of the head. The inclusion of both phantoms enables the evaluation of reconstruction performance across structurally distinct image types and under varying noise conditions.

To systematically evaluate both the convergence characteristics and the perceptual quality of the reconstructed images, we employed several quantitative metrics. First, convergence behavior was assessed using the root mean squared error E(z) between the reconstructed image *z* and the noise-free ground truth $x^{*}$, defined as

(11) $$E\left(z\right):={\left(\sum_{j=1}^{J}{\left(x_{j}^{*}-z_{j}\right)}^{2}\right)}^{\frac{1}{2}},$$

where *J* denotes the total number of pixels. A decreasing trend in E(z(k)) across iterations indicates improved fidelity to the reference image.

In addition to E(z), we evaluated image quality using the Peak Signal-to-Noise Ratio [23,24] (PSNR) and the Multi-Scale Structural Similarity Index [25] (MS-SSIM). PSNR, commonly used in image processing tasks, was calculated as follows:

(12) $$\mathrm{PSNR}=10\log_{10}\left(\frac{\max^{2}\cdot J}{E{\left(z\right)}^{2}}\right),$$

where max denotes the maximum possible pixel intensity. Higher PSNR values generally correspond to lower distortion, although the metric does not always align with perceived image quality [26]. To address this limitation, we also computed MS-SSIM, a perception-based metric that incorporates image structure at multiple resolutions. MS-SSIM is an improved version of SSIM [27] that evaluates image quality across multiple scales and is known to provide a more accurate assessment of perceptual image quality compared to SSIM. It is defined as follows:

(13) $$\mathrm{MS}-\mathrm{SSIM}\left(x^{*},z\right)={\left[l_{M}\left(x^{*},z\right)\right]}^{\mu_{M}}\cdot \prod_{m=1}^{M}{\left[c_{m}\left(x^{*},z\right)\right]}^{\nu_{m}}\cdot {\left[s_{m}\left(x^{*},z\right)\right]}^{\xi_{m}}.$$

Here, $l_{M}$, $c_{m}$, and $s_{m}$ represent luminance, contrast, and structural similarity components at each scale *m*, respectively. The exponents $\mu_{M}$, $\nu_{m}$, and $\xi_{m}$ are predefined weights based on human visual sensitivity, consistent with the configuration proposed in [25].

### 5.2. Experiments with Clinical SPECT Data

To further assess the practical applicability of the proposed GREM algorithm, we conducted experiments using clinical SPECT data acquired from a Technetium-99m Galactosyl Human Serum Albumin (^99m^Tc-GSA) liver scintigraphy examination. The data were obtained using a Symbia Pro.specta SPECT/CT system (Siemens Healthcare GmbH, Forchheim, Germany) at a clinical site. The imaging protocol employed a matrix size of 128×128 pixels per slice and continuous acquisition over 180 degrees using dual-head detectors. Projection data were acquired every 6 degrees, resulting in a total of 60 projection angles. Low-energy high-resolution (LEHR) collimators were used for all acquisitions.

**Figure 3:**
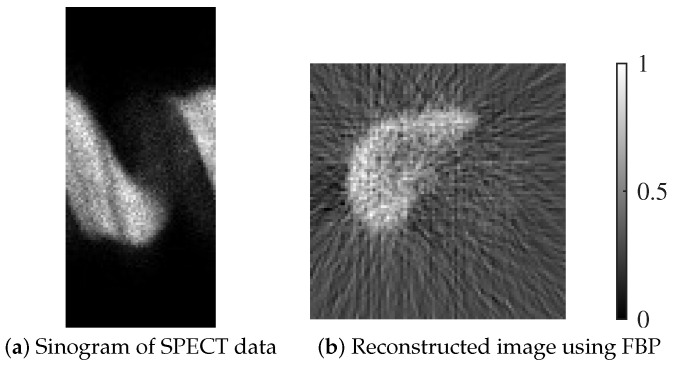
Example of GSA liver scintigraphy data: (**a**) Sinogram obtained from SPECT acquisition. (**b**) Corresponding reconstructed image using FBP.

Figure 3 illustrates an example of the clinical data used in our experiments. Figure 3a shows the raw sinogram obtained from a ^99m^Tc-GSA liver SPECT acquisition using a dual-head detector system. Figure 3b presents the corresponding reconstructed image obtained by applying the conventional filtered back projection (FBP) method.

This protocol, commonly utilized in hepatobiliary scintigraphy, enables the visualization of functional liver tissue based on the receptor-binding properties of GSA. To ensure a fair comparison among reconstruction algorithms, raw projection data were used as acquired directly from the planar detectors, without the application of standard attenuation or scatter corrections. These unprocessed data were reconstructed using the proposed GREM algorithm, as well as the two baseline methods (MLEM and MLEM-TV).

Since the ground truth distribution is not accessible in clinical data, quantitative evaluation was performed using indirect image-based metrics. Specifically, density profiles extracted along representative linear paths were analyzed to evaluate contrast preservation and the smoothness of signal transitions. These metrics provide a practical basis for comparing reconstruction performance under realistic clinical conditions in which direct error measurements are not feasible. For all reconstruction methods, the initial image estimate was defined as a uniform positive image derived from the measured projection data, given by

(14) $$x_{j}^{0}=\frac{\sum_{i=1}^{I}p_{i}}{\sum_{i=1}^{I}\sum_{j=1}^{J}h_{ij}},$$

for all pixels *j*. The same initial estimate was used for all compared methods (MLEM, MLEM-TV, and GREM) to ensure a fair and consistent comparison.

## 6. Results

This section presents the experimental results obtained using the numerical phantoms (Shepp–Logan and digitized Hoffman) and the clinical SPECT data.

### 6.1. Results with Numerical Phantoms

#### 6.1.1. Shepp–Logan Phantom

**Figure 4:**
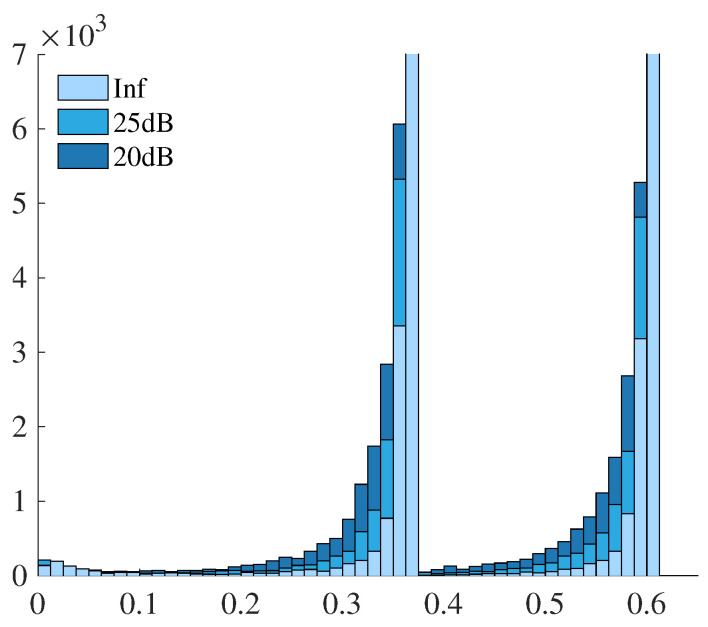
Histograms of graph edge weights computed from the Shepp–Logan phantom under different noise levels (noise-free (Inf), 25 dB, and 20 dB).

Before evaluating the convergence characteristics, we investigate how the introduction of the graph structure reflects the differences caused by noise in terms of edge information. Specifically, we examine the distribution of the edge weights computed by Equation (8) for the Shepp–Logan phantom under three noise conditions: noise-free (Inf), 25 dB, and 20 dB. Figure 4 presents the histograms of the edge weights. The horizontal axis indicates the weight values, and the vertical axis shows the corresponding counts. The horizontal and vertical ranges have been adjusted to improve visual clarity.

The histograms clearly demonstrate that in the noise-free case, edge weights tend to concentrate around specific high values, reflecting the smooth and homogeneous regions within the phantom. In contrast, as the noise level increases, the histogram becomes broader and flatter, indicating a wider variation in edge weights. This increased variability in the edge weights reflects the influence of noise on the local intensity structure, which directly affects the construction of the graph Laplacian used in the reconstruction term. These observations indicate that the graph formulation effectively captures image noise characteristics as structural information in the graph representation.

**Figure 5:**
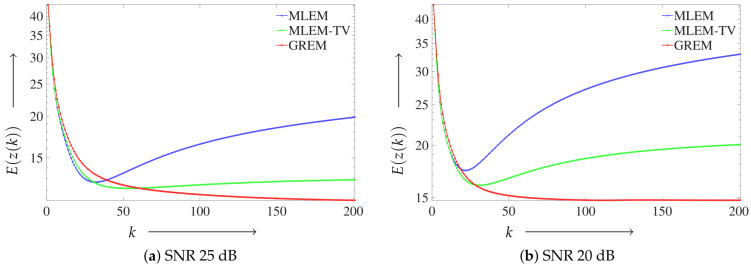
Convergence behavior of the reconstruction algorithms for the Shepp–Logan phantom under different noise levels: (**a**) Result at SNR 25 dB. (**b**) Result at SNR 20 dB.

Next, we present the convergence characteristics of the proposed method using the Shepp–Logan phantom. Figure 5 illustrates the convergence behavior, where the vertical axis shows the value of E(z(k)) defined in Equation (11), and the horizontal axis corresponds to the number of iterations. Figure 5a corresponds to the result at SNR 25 dB and Figure 5b to that at SNR 20 dB. In each plot, the performance of the conventional MLEM, the regularized MLEM-TV, and the proposed GREM method is presented for comparison.

**Figure 6:**
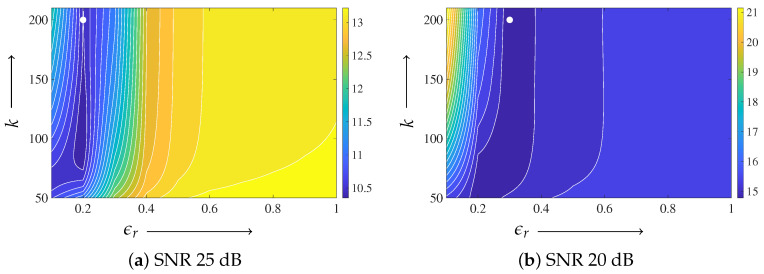
Parameter-plane visualization for the Shepp–Logan phantom with the iteration number on the vertical axis and $\epsilon_{r}$ on the horizontal axis, with $\epsilon_{d}=1.0$ fixed. (**a**) SNR 25 dB, (**b**) SNR 20 dB. Only iterations from k=50 onward are displayed. The color represents the value of the evaluation function E(z(k)). The filled white circle indicates the minimum value at the final iteration k=200.

For the Shepp–Logan phantom, the parameter $\epsilon_{d}$ was fixed to 1.0. Based on the parameter-plane analysis shown in Figure 6, $\epsilon_{r}$ was set to 0.2 for the 25 dB case and to 0.3 for the 20 dB case. The regularization parameter in (10) was fixed to γ=0.01 for both noise levels. To reduce the degrees of freedom and to provide a consistent baseline, the regularization weight in MLEM-TV was also fixed to 0.01, which is of the same order as γ, and this value was used consistently in all experiments.

The proposed GREM method demonstrated the best convergence at both 25 and 20 dB noise levels, with particularly noticeable improvement at the higher noise level of 20 dB. In fact, it achieved the lowest evaluation function values among all the algorithms tested.

**Figure 7:**
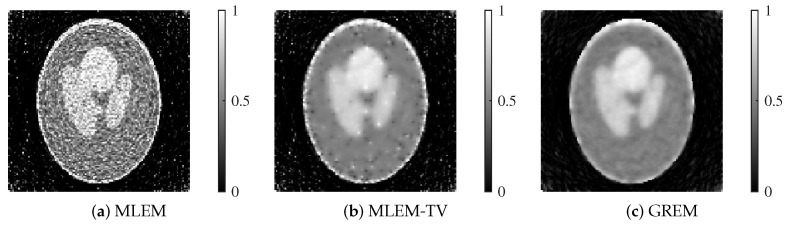
Reconstructed images of MLEM, MLEM-TV, and GREM at a noise level of 25 dB: (**a**) MLEM, (**b**) MLEM-TV, (**c**) GREM.

**Figure 8:**
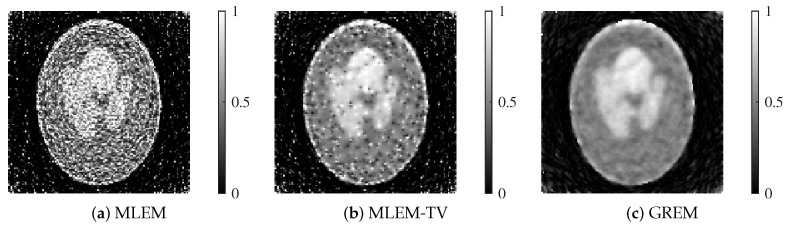
Reconstructed images of MLEM, MLEM-TV, and GREM at a noise level of 20 dB: (**a**) MLEM, (**b**) MLEM-TV, (**c**) GREM.

We present the reconstructed images by each method in Figure 7 and Figure 8.

Each reconstructed image shown corresponds to the result after 200 iterations. At both 25 dB and 20 dB noise levels, MLEM exhibits noticeable noise across the image. While MLEM-TV reduces noise at 25 dB, it suffers from excessive smoothing, resulting in a loss of fine details. Furthermore, at 20 dB, MLEM-TV also presents significant noise, similar to MLEM. In contrast, the proposed method successfully balances noise suppression and edge preservation at both noise levels, maintaining high image quality even under severe noise conditions.

Table 1 summarizes the quantitative evaluation results in terms of PSNR and MS-SSIM metrics.

From the table, it is observed that the proposed GREM method achieved the highest PSNR and MS-SSIM values under both noise levels. Specifically, at 25 dB, GREM yielded a PSNR of 21.169 and an MS-SSIM of 0.869, outperforming both MLEM and MLEM-TV. Even under the higher noise level of 20 dB, GREM maintained superior performance with a PSNR of 18.744 and an MS-SSIM of 0.787.

Regarding computational time, as a concrete example, in the Shepp–Logan numerical phantom experiment, the total execution time was approximately 10.5 s for MLEM, 18.6 s for MLEM-TV, and 54.3 s for GREM. These measurements were obtained on an Apple M4 chip (Apple Inc., Cupertino, CA, USA) with 24 GB memory using MATLAB R2025b (MathWorks, Natick, MA, USA) with the same number of iterations and the same initialization. While the asymptotic per-iteration complexity is comparable among the methods, the additional cost in GREM mainly originates from per-iteration graph weight computation and Laplacian interactions in the present implementation. In our MATLAB implementation, the TV step is computed efficiently, whereas GREM incurs additional overhead from graph construction. Note that these runtimes are implementation-dependent and reflect the current MATLAB implementations rather than optimized code for each method; whether this overhead is acceptable depends on the application and desired image quality.

#### 6.1.2. Digitized Hoffman Phantom

**Figure 9:**
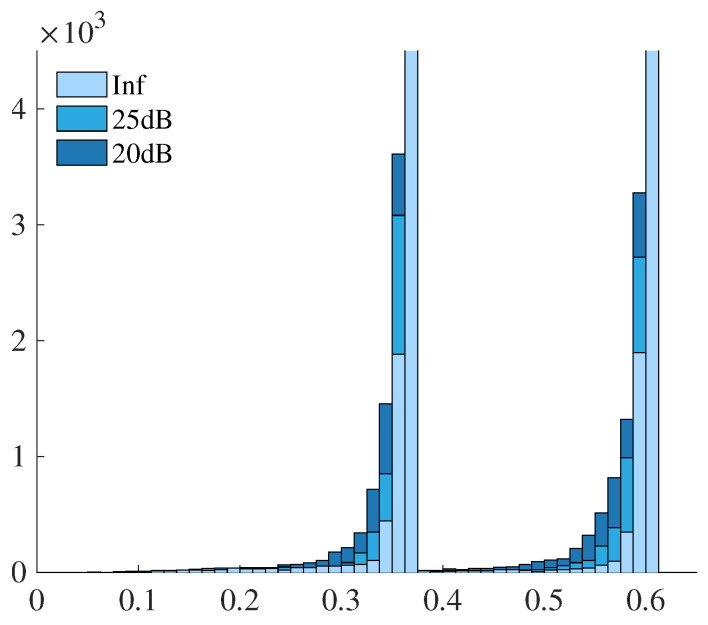
Histograms of graph edge weights computed from the digitized Hoffman phantom under different noise levels (noise-free (Inf), 25 dB, and 20 dB).

The results for the digitized Hoffman phantom are also presented. As with the Shepp–Logan phantom, histograms of the graph edge weights were computed under noise-free, 25 dB, and 20 dB conditions. The graph weights were constructed based on Equation (8), and the resulting edge weight distributions are shown in Figure 9. The horizontal axis denotes the edge weight values, and the vertical axis represents the count, with horizontal and vertical scaling adjusted for better visibility.

A similar trend is observed, where the histograms become more dispersed as the noise level increases. This consistency across different phantom types indicates that the graph representation effectively captures the influence of image noise in the edge structure, regardless of the underlying image content.

**Figure 10:**
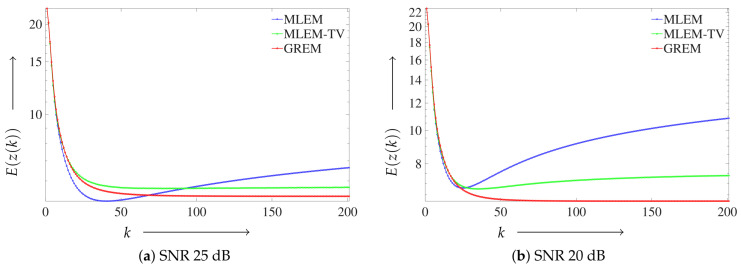
Convergence behavior of the reconstruction algorithms for the digitized Hoffman phantom under different noise levels: (**a**) Result at SNR 25 dB. (**b**) Result at SNR 20 dB.

Subsequently, the convergence behavior is examined, as shown in Figure 10.

**Figure 11:**
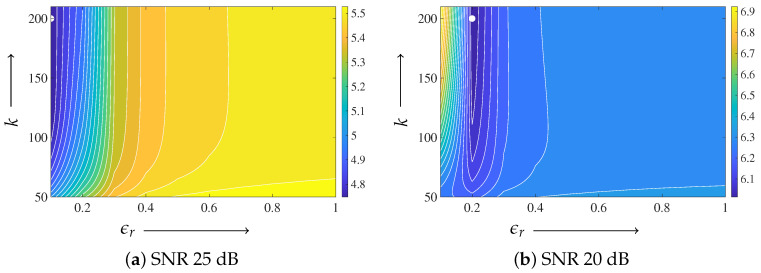
Parameter-plane visualization for the digitized Hoffman phantom with the iteration number on the vertical axis and $\epsilon_{r}$ on the horizontal axis, with $\epsilon_{d}=1.0$ fixed. (**a**) SNR 25 dB, (**b**) SNR 20 dB. Only iterations from k=50 onward are displayed. The color represents the value of the evaluation function E(z(k)). The filled white circle indicates the minimum value at the final iteration k=200.

For the digitized Hoffman phantom, the parameter $\epsilon_{d}$ was fixed to 1.0 for both noise levels, following the same strategy as that used for the Shepp–Logan phantom. Figure 11 shows the parameter-plane visualization of the evaluation function E(z(k)) as a function of the iteration number *k* and the parameter $\epsilon_{r}$. From this analysis, the minimum value at the final iteration k=200 was observed at $\epsilon_{r}=0.1$ for the 25 dB case and at $\epsilon_{r}=0.2$ for the 20 dB case. Accordingly, these values were adopted in the subsequent reconstruction experiments. The regularization parameter γ was fixed to 0.01 in both experiments.

Although the digitized Hoffman phantom exhibits a more complex structure than the Shepp–Logan phantom, the proposed GREM method still demonstrated the best convergence behavior.

**Figure 12:**
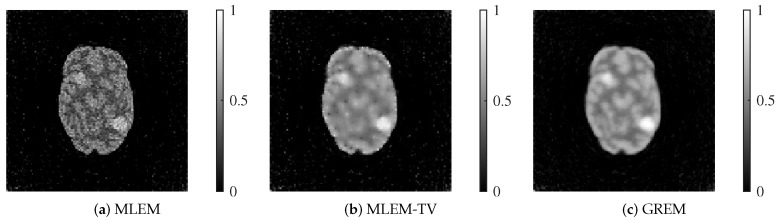
Reconstructed images of MLEM, MLEM-TV, and GREM at a noise level of 25 dB: (**a**) MLEM, (**b**) MLEM-TV, (**c**) GREM.

**Figure 13:**
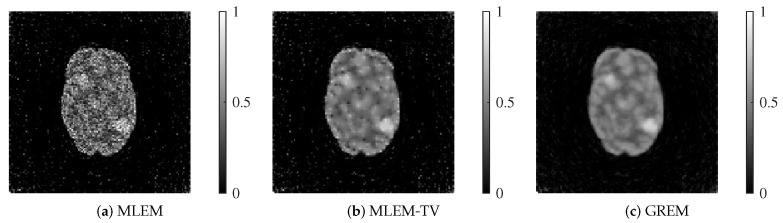
Reconstructed images of MLEM, MLEM-TV, and GREM at a noise level of 20 dB: (**a**) MLEM, (**b**) MLEM-TV, (**c**) GREM.

As shown in Figure 12 and Figure 13, the reconstructed images highlight the superior performance of the proposed GREM method. Each reconstructed image shown corresponds to the result after 200 iterations. In both 25 dB and 20 dB noise conditions, GREM clearly outperforms MLEM and MLEM-TV in terms of preserving details and reducing noise. While MLEM shows significant noise presence in the images, and MLEM-TV exhibits excessive smoothing at 25 dB, GREM effectively balances noise reduction with edge preservation, even under the higher noise levels. This qualitative assessment is consistent across both noise conditions, where GREM produces the clearest and most accurate reconstructions.

Further supporting these visual observations, Table 2 presents the quantitative results, demonstrating that GREM achieves the highest PSNR and MS-SSIM values in comparison to MLEM and MLEM-TV. Specifically, GREM surpasses both methods in performance under both 25 dB and 20 dB noise conditions, indicating its robustness in handling varying levels of noise. These quantitative metrics reinforce the visual quality of the images, confirming that GREM offers superior reconstruction performance across both qualitative and quantitative measures.

### 6.2. Results with Clinical SPECT Data

For the ^99m^Tc-GSA liver SPECT data, a parameter-plane analysis analogous to that used in the numerical phantom experiments cannot be performed because the ground-truth activity distribution is unavailable and the evaluation function E(z(k)) in (11) cannot be defined. Therefore, the graph parameters were selected by referencing the numerical phantom experiments, aiming to use values that lie in a stable low-error region and avoid excessive smoothing.

**Figure 14:**
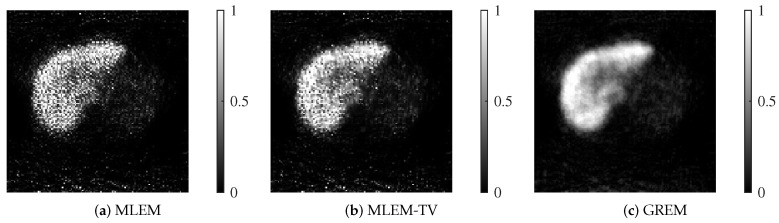
Reconstructed images of MLEM, MLEM-TV, and GREM with ^99m^Tc-GSA liver SPECT data: (**a**) MLEM, (**b**) MLEM-TV, (**c**) GREM.

Specifically, we fixed $\epsilon_{d}=1.0$ and set $\epsilon_{r}=0.3$, which is within the range of $\epsilon_{r}$ values that provided stable performance in the phantom studies under comparable noise conditions. The regularization parameter in (10) was fixed to γ=0.01. The reconstructed images for clinical SPECT data are shown in Figure 14.

The reconstructed images are displayed after normalizing the count values for better observation. Each reconstructed image shown corresponds to the result after 50 iterations.

**Figure 15:**
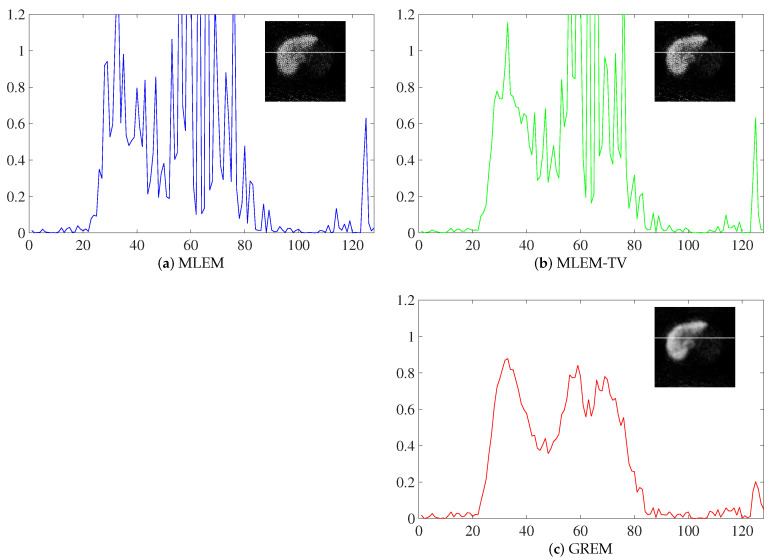
Reconstructed images of MLEM, MLEM-TV, and GREM with ^99m^Tc-GSA liver SPECT data. The vertical axis of the concentration profile is displayed with a maximum value set to 1.2. (**a**) MLEM, (**b**) MLEM-TV, (**c**) GREM.

Additionally, the concentration profile images at arbitrary positions are shown in Figure 15.

In the concentration profile plots, the location where the profiles were extracted is highlighted on the corresponding reconstructed images. The concentration profiles were obtained from the same location across all methods, allowing for a direct comparison of the performance of MLEM, MLEM-TV, and the proposed GREM method.

The GREM method clearly demonstrates better preservation of edges and less noise compared to MLEM and MLEM-TV. This is particularly evident in the profile plots, where the GREM method maintains a more accurate and consistent intensity distribution, indicating superior image reconstruction quality.

## 7. Discussion

In this study, we proposed GREM, a graph-enhanced multiplicative extension of the standard MLEM algorithm. By augmenting the MLEM update with a graph-based coupling term defined through the Laplacian L=D−W, the proposed method incorporates spatial proximity and intensity similarity among neighboring pixels. This design encourages consistency within locally homogeneous regions while allowing transitions across weakly connected areas, thereby suppressing noise-induced fluctuations without excessive oversmoothing.

The performance of GREM was evaluated using two numerical phantoms (Shepp–Logan and digitized Hoffman) under moderate and high noise conditions (SNR 25 dB and 20 dB). At a fixed iteration number, GREM consistently achieved higher PSNR and MS-SSIM than MLEM and MLEM-TV, with more pronounced improvements observed in the higher-noise case. These results indicate that incorporating graph-based neighborhood information can improve both perceptual quality and quantitative fidelity in low-count regimes where standard MLEM tends to amplify noise and TV-regularized reconstruction may suffer from oversmoothing or parameter sensitivity.

The proposed method was also applied to clinical ^99m^Tc-GSA liver SPECT data. Visual assessment of reconstructed images and comparison of representative one-dimensional intensity profiles suggest that GREM provides clearer structural delineation with reduced noise compared with MLEM and MLEM-TV under identical initialization and iteration counts. Because ground-truth activity distributions are not available for clinical data, these observations should be interpreted as empirical evidence of improved image quality rather than direct error reduction.

From a theoretical standpoint, the main property retained by the proposed iteration is preservation of non-negativity. Under standard assumptions ($h_{ij}\geq 0$, $p_{i}\geq 0$, $c_{j}=\sum_{i}h_{ij}>0$, $W_{jj^{\prime }}\geq 0$, γ≥0), the multiplicative structure of the update guarantees that all iterates remain in $R_{+}^{J}$ for any non-negative initialization. This property is essential in emission tomography, where reconstructed activity distributions must remain physically meaningful. In practice, a small positive floor is applied to ${\left(Hz\right)}_{i}$ to avoid division by zero in low-count bins, without affecting the non-negativity argument.

The edge-weight histograms provide insight into the influence of noise on the graph structure. In noise-free cases, weights tend to concentrate near larger values, reflecting locally homogeneous regions. As noise increases, the weight distributions broaden, indicating increased heterogeneity in local intensity differences. This behavior is consistent with the role of the graph term as an adaptive neighborhood interaction that balances smoothing within homogeneous regions and preservation of structural transitions.

Parameter-plane analyses reveal a valley-shaped stable region for small $\epsilon_{r}$ with $\epsilon_{d}=1.0$ fixed, indicating that the performance is robust over a range of parameter values rather than being tuned to a single point. The same parameter ranges were effective across different noise levels and image structures, suggesting that moderate variations in acquisition conditions do not require strict re-tuning. Possible strategies for automatic parameter adjustment include noise-level-dependent scaling based on projection statistics and adaptive updates guided by simple image smoothness or residual measures during the iteration, which can be incorporated within the same update rule without changing the overall framework.

One limitation of the present implementation is the use of a fixed local neighborhood, and extension to more flexible graph constructions remains a topic for future investigation.

Comparisons in this study were limited to MLEM and TV-regularized MLEM in order to focus on training-free, model-based EM-type reconstruction methods that preserve non-negativity by construction. Although recent data-driven and hybrid approaches have shown strong performance, the proposed GREM framework is intended as a complementary model-based alternative rather than a direct competitor to learning-based methods.

## 8. Conclusions

We presented GREM, a graph-enhanced multiplicative reconstruction algorithm for SPECT imaging. By incorporating a graph-based coupling term into an MLEM-type update, the proposed method improves empirical image quality in both numerical phantom studies and clinical ^99m^Tc-GSA liver SPECT data, particularly under higher noise conditions, while preserving non-negativity of the iterates. The proposed framework does not rely on external training data and provides a practical approach to edge-aware noise suppression through graph-structured neighborhood interactions.

## Figures and Tables

**Table 1:** Results of quantitative indicators.

	Method	SNR 25 dB	SNR 20 dB
PSNR	MLEM	16.176	11.775
	MLEM-TV	19.928	16.087
	GREM	21.169	18.744
MS-SSIM	MLEM	0.762	0.641
	MLEM-TV	0.831	0.705
	GREM	0.869	0.787

**Table 2:** Results of quantitative indicators.

	Method	SNR 25 dB	SNR 20 dB
PSNR	MLEM	25.694	21.439
	MLEM-TV	27.010	24.761
	GREM	28.629	27.604
MS-SSIM	MLEM	0.923	0.836
	MLEM-TV	0.920	0.851
	GREM	0.942	0.894

## Data Availability

The original contributions presented in this study are included in the article. Further inquiries can be directed to the corresponding author.
